# Synthesis of Some Novel Chromenopyrimidine Derivatives and Evaluation of Their Biological Activities 

**Published:** 2014

**Authors:** Akbar Mobinikhaledi, Naser Foroughifar, Tahere Mosleh, Ahmad Hamta

**Affiliations:** a *Department of Chemistry, Faculty of Science, Arak University, Arak 38156-8-8349, Iran. *; b *Faculty of Chemistry, Tehran North Branch, Islamic Azad University, Tehran, Iran. *; c *Department of Biology, Arak University, Arak 38156-8-8349, Iran. *

**Keywords:** Imine, Chromenopyrimidine, Pyranopyrimidine, Antibacterial, Multicomponent

## Abstract

Pyrimidine nucleosides are constituents of fundamental structure of the cells. There has been considerable attentions in the chemistry of pyrimidine derivatives due to having a wide range of biological activities such as antiviral, anti-malarial agents, cytostatic, antithelemintic, antibacterial, adenosine receptor ligands, anti-cancer agents, compounds targeting delayed-type hypersensivity and anti-convulsant agents. As a part of our research work in the synthesis of pyrimidines containing biological activities, a series of chromenopyrimidine derivatives were synthesized by reaction of an intermediate imine and ammonia derivatives in good to high yields. All synthesized compounds were characterized using IR and NMR (^1^H and ^13^C) spectroscopy and elemental analysis data. The antibacterial activity of these compounds was investigated against Staphylococcus aureus (RTCC, 1885), and Escherichia Coli (ATCC, 35922).

## Introduction

Pyrimidine is a basic nucleus in DNA and RNA and plays an essential role in chemistry and biological systems (1). Pyrimidine derivatives have received considerable attentions due to their diverse range of therapeutic and pharmacological properties as antiviral (2), cytostatic (3-5), immunomodulating and antibacterial (6-9). Fused heterocyclic pyrimidines have also shown a wide range of biological activities, such as antitumor, antiviral, antimicrobial, antibacterial and anti-inflammatory (10-17). Study of various substituted pyrimidine derivatives indicated a good correlation between compound structures and antibacterial activity (18-24). Several methods have been reported for the synthesis of simple pyrimidine derivatives (1, 25-28). However, because of the incessant interest in this field, new efficient synthesis of some fused pyrimidine derivatives is still an important objective for synthetic organic chemists in order to find compounds with different biological activities. 

In view of these reports and also due to continuation of our interests on synthesis of pyrimidines (29-32), we wish to report synthesis of some fused pyrimidine derivatives. 

## Experimental

*Materials*

All reagents and solvents used are commercially available. Reactions were monitored by thin layer chromatography (TLC) using silica gel F_254_ aluminum sheets (Merck). Melting points were measured on an Electrothermal apparatus. Infra-red spectra were recorded (KBr discs) with a Galaxy Series FT-IR 5000 spectrometer. The ^1^H NMR and ^13^C NMR spectra were recorded with a Bruker Avance 300 MHz spectrometer with DMSO-*d*_6_ and CDCl_3_ as the solvent and tetramethylsilane as an internal standard. Microanalyses were performed by the Elemental Analyzer (Elemental, Vario EL III) at the Arak University. The microbial strains are identified strains and were obtained from the Pasteur Institute of Iran. The bacterial strains studied are Staphylococcus aureus (RTCC, 1885), and Escherichia Coli (ATCC, 35922). 

*General procedure for the synthesis of compounds 6-8*

In a typical experimental procedure (34-36), benzaldehyde 1 (1 mmol), malononitrile 2 (1 mmol), dimedone 3, resorcinol 4 or 2-naphthol 5 (1 mmol) were mixed in solvent and triethylamine (2-3 drops) as a catalyst was added. The reaction mixture was refluxed for 2-4 h. After the completion of the reaction, it was filtered and recrystallized from ethanol to afford the pure product 6-8.

2-Amino-7,7-dimethyl-5-oxo-4-phenyl-5,6,7,8-tetrahydro-4*H*-chromene-3-carbonitrile (6): Yield: 85% m.p 227-229 °C. IR (KBr): 3395, 3326, 2199, 1682 cm^-1^. Elemental analysis. Found, %: C 73.52; H 6.28; N 9.36, C_18_H_18_N_2_O_2_, Calculated, %: C 73.45; H 6.16; N 9.52. 

2-Amino-7-hydroxy-4-phenyl-4*H*-chromene-3-carbonitrile (7): Yield: 78%, m.p 234-235 °C. IR (KBr): 3499, 3427, 3331, 2193 cm^-1^. Elemental analysis. Found, %: C 72.48; H 4.61; N 10.7. C_16_H_12_N_2_O_2_, Calculated, %: C 72.72; H 4.58; N 10.60. 

3-Amino-1-phenyl-1*H*-benzo[*f*]chromene-2-carbonitrile (8): Yield: 80%, m.p 279-280 °C. IR (KBr): 3435, 3338, 2183 cm^-1^. Elemental analysis. Found. %: C 80.27; H 4.50; N 9.74. Calculated, %: C_20_H_14_N_2_O, C 80.52; H 4.73; N 9.39. 

*General procedure for the synthesis of compounds 9-11*

To a solution of 2-amino-3-carbonitrile 6-8 (1 mmol) in 1,4-dioxane (20 mL) was added triethylorthoformate (2 mmol) and acetic anhydride (2 mmol). The reaction mixture was heated under reflux condition for 2-4 h. After the completion of the reaction, the solvent was removed and the precipitate was recrystallized from ethanol to afford the pure product 9-11. 

Ethyl N-3-cyano-7,7-dimethyl-5-oxo-4-phenyl-5,6,7,8-tetrahydro-4*H*-chromen-2-ylformimidate (9): reaction time: 2h, Yield 85%, m.p 178-180 °C. IR (KBr): 2206, 1674 cm^-1^. ^1^H N.M.R. (CDCl_3_) (ppm) (*J*, Hz): 1.11 (3H, s, CH_3_), 1.19 (3H, s, CH_3_), 1.28 (3H, t, CH_3_, *J*=6.6), 2.25 (2H, s, CH_2_), 2.47 (2H, s, CH_2_), 4.37 (2H, q, *J*=6.6, CH_2_,) 4.54 (1H, s, H_pyran_), 7.21-7.35 (5H, m, H_aromatic_), 8.25 (1H, s, H_imine_). Elemental analysis. Found. %: C 72.27; H 6.14; N 7.89. C_21_H_22_N_2_O, Calculated, %: C 71.98; H 6.33; N 7.99. 

Ethyl N-3-cyano-7-hydroxy-4-phenyl-4*H*-chromen-2-ylformimidate (10): reaction time: 3h, Yield 80%, m.p 166-168 °C. IR (KBr): 3110, 2224 cm^-1^. ^1^H N.M.R. (CDCl_3_) (ppm) (*J*, Hz): 1.34 (3H, t, *J*=5.6, CH_3_,), 4.39 (2H, q, *J*=5.6, CH_2_,), 4.83 (1H, s, H_pyran_), 6.79-7.37 (8H, m, H_aromatic_ and 1H, OH), 8.39 (1H, s, H_imine_). ^13^C N.M.R. (CDCl_3_) (ppm): 21.1, 42.5, 64.2, 81.2, 110.2, 119.4, 119.7, 127.3, 127.7, 127.9, 128.1, 128.2, 129.0, 143.3, 157.0, 159.6, 169.1. Elemental analysis. Found, %: C 71.01; H 5.21; N 8.51. C_19_H_16_N_2_O_3_, Calculated, %: C 71.24; H 5.03; N 8.74. 

Ethyl N-2-cyano-1-phenyl-1*H*-benzo[*f*]chromen-3-ylformimidate (11): reaction time: 4 h, Yield 75%, m.p 224-226 °C. IR (KBr): 2224 cm^-1^. ^1^H N.M.R. (CDCl_3_) (ppm) (*J*, Hz): 1.35 (3H, t, *J*=7.1, CH_3_), 4.40 (2H, q, *J*=7.1, CH_2_,), 5.30 (1H, s, H_pyran_), 7.21-7.86 (11H, m, H_aromatic_), 8.45 (1H, s, H_imine_). ^13^C N.M.R. (CDCl_3_) (ppm): 13.9, 40.8, 64.2, 82.2, 113.9, 116.8, 118.3, 123.7, 125.2, 127.4, 127.7, 128.6, 129.1, 129.9, 130.7, 131.5, 143.1, 147.6, 156.6, 159.5. 168.2. Elemental analysis. Found. %: C 77.76; H 5.02; N 9.021. C_23_H_18_N_2_O_2_, Calculated, %: C 77.95; H 5.12; N 9.03. 

*General procedure for the synthesis of compounds 12-20*

A mixture of imine 9-11 (1 mmol) and ammonia or primary amine (1 mmol) in ethanol or 1,4-dioxane (15 mL) was refluxed for the indicated time (Table 1). After completion of the reaction, the solid material was separated and recrystallized from ethanol to give compounds 12-20.

4-Imino-8,8-dimethyl-5-phenyl-5,7,8,9-tetrahydro-3*H*-chromeno[2,3-*d*]pyrimidin- 6(4*H*)-one (12): IR (KBr): 3308-3464, 1691 cm^-1^. ^1^H N.M.R. (DMSO-d_6_) (ppm) (*J*, Hz): 0.90-1.10 (6H, s, 2CH_3_), 2.14 (2H, s, CH_2_), 2.35 (2H, s, CH_2_), 5.58 (1H, s, H_pyran_), 6.90-7.31 (6H, m, H_aromatic_), 7.81 (1H, s, NH), 8.07 (1H, s, H_Iimine_), 11.61 (1H, s, NH). ^13^C N.M.R. (DMSO-d6) (ppm): 26.9, 28.5, 43.6, 50.5, 56.5, 127.0, 127.9, 128.5, 140.9, 143.5, 147.7, 156.7, 163.1, 164.4, 165.1, 193.8. Elemental analysis. Found. %: C 71.27; H 5.70; N 13.28. C_19_H_19_N_3_O_2_, Calculated, %: C 71.01; H 5.96; N 13.08. 

4-Imino-8,8-dimethyl-3-(3-nitrophenyl)-5-phenyl-5,7,8,9-tetrahydro-3*H*-chromeno[2,3-*d*]pyrimidin-6(4*H*)-one (13): IR (KBr): 3421, 1684, 1645 cm^-1^. ^1^H N.M.R. (DMSO-d_6_) (ppm) (*J*, Hz): 1.00 (3H, s, CH_3_), 1.08 (3H, s, CH_3_), 2.13 (2H, s, CH_2_), 2.64 (2H, s, CH_2_), 4.40 (1H, s, H_pyran_), 7.21-9.00 (11H, m, H_aromatic_, H_imine_, NH). ^13^C N.M.R. (DMSO-d_6_) (ppm): 25.1, 25.4, 27.3, 29.8, 31.2, 52.6, 101.2, 123.7, 124.9, 126.1, 128.7, 129.9, 131.2, 133.7, 135.1, 139.5, 142.1, 143.9, 145.1, 148.7, 153.2, 159.9, 173.4. Elemental analysis. Found. %: C 67.62; H 4.91; N 12.50. C_25_H_22_N_4_O_4_, Calculated, %: C 67.86; H 5.01; N 12.66. 

4-Imino-5-phenyl-4,5-dihydro-3*H*-chromeno[2,3-*d*]pyrimidin-8-ol (14): IR (KBr): 3462, 3310, 3094 cm^-1^. ^1^H N.M.R. (DMSO-d_6_) (ppm) (*J*, Hz): 5.15 (1H, s, H_pyran_), 6.51-7.25 (10H, m, H_aromatic_, NH), 8.09 (1H, s, H_imine_), 9.65 (1H, s, OH). ^13^C N.M.R. (DMSO-d_6_) (ppm): 37.9, 96.6, 103.5, 112.8, 115.9, 127.1, 127.7, 129.0, 130.2, 145.4, 150.5, 156.9, 157.7, 162.9, 163.1. Elemental analysis. Found, %: C 70.23; H 4.63; N 14.59. C_17_H_13_N_3_O_2_, Calculated, %: C 70.09; H 4.50; N 14.42. 

3-(2-Chlorophenyl)-4-imino-5-phenyl-4,5-dihydro-3*H*-chromeno[2,3-*d*]pyrimidin-8-ol (15): IR (KBr): 3393, 3157, 1635 cm^-1^. ^1^H N.M.R. (DMSO-d_6_) (ppm) (*J*, Hz): 5.48 (1H, s, H_pyran_), 6.56-8.26 (14H, m, H_aromatic_, NH, H_imine_), 9.79 (1H, s, OH). ^13^C N.M.R. (DMSO-d_6_) (ppm): 37.7, 98.9, 103.5, 113.1, 115.3, 126.8, 127.5, 127.9, 128.6, 129.3, 129.9, 130.4, 136.2, 144.8, 150.1, 156.7, 157.8, 159.82, 159.83, 163.3. Elemental analysis. Found, %: C 68.89; H 4.21; Cl 8.61; N 10.29. C_23_H_16_ClN_3_O_2_, Calculated, %: C 68.74; H 4.01; Cl 8.82; N 10.46. 

4-Imino-3-(3-nitrophenyl)-5-phenyl-4,5-dihydro-3*H*-chromeno[2,3-*d*]pyrimidin-8-ol (16): IR (KBr): 3402, 3142, 1612 cm^-1^. ^1^H N.M.R. (DMSO-d_6_) (ppm) (*J*, Hz): 5.74 (1H, s, H_pyran_), 6.60-8.05 (12H, m, H_aromatic_), 8.60 (1H, s, H_imine_), 8.95 (1H, s, NH), 9.76 (1H, s, OH). ^13^C N.M.R. (DMSO-d_6_) (ppm): 37.2, 100.4, 103.7, 107.6, 113.3, 115.3, 117.6, 120.4, 127.3, 127.5, 129.3, 130.1, 130.2, 141.2, 145.3, 148.3, 150.3, 156.5, 158.0, 159.0, 163.6. Elemental analysis. Found, %: C 66.76; H 3.84; N 13.35. C_23_H_16_N_4_O_4_, Calculated, %: C 66.99; H 3.91; N 13.59. 

4-Imino-5-phenyl-3-p-tolyl-4,5-dihydro-3*H*-chromeno[2,3-*d*]pyrimidin-8-ol (17): IR (KBr): 3427, 3092, 1635 cm^-1^. ^1^H N.M.R. (DMSO-d_6_) (ppm) (*J*, Hz): 2.24 (3H, s, CH_3_), 5.63 (1H, s, H_pyran_), 6.56-7.41 (12H, m, H_aromatic_), 8.29 (1H, s, H_imine_), 8.31 (1H, s, NH), 9.71 (1H, s, OH). ^13^C N.M.R. (DMSO-d_6_) (ppm): 20.9, 37.2, 99.0, 103.5, 113.1, 115.5, 122.1, 127.3, 127.6, 129.2, 129.3, 130.2, 132.7, 137.2, 145.4, 150.3, 156.6, 157.8, 159.4, 163.1. Elemental analysis. Found. %: C 75.69; H 4.91; N 11.26. C_24_H_19_N_3_O_2_, Calculated, %: C 75.57; H 5.02; N 11.02. 

3-(4-Ethylphenyl)-4-imino-5-phenyl-4,5-dihydro-3*H*-chromeno[2,3-*d*]pyrimidin-8-ol (18): IR (KBr): 3421, 3109, 1635 cm^-1^. ^1^H N.M.R. (DMSO-d_6_) (ppm) (*J*, Hz): 1.13 (3H, t, *J*=7.0, CH_3_), 2.49 (2H, q, *J*=7.0, CH_2_), 5.63 (1H, s, H_pyran_), 6.03-7.42 (12H, m, H_aromatic_), 8.29 (1H, s, H_imine_), 8.35 (1H, s, NH), 9.76 (1H, s, OH). ^13^C N.M.R. (DMSO-d_6_) (ppm): 16.2, 28.0, 37.1, 99.0, 103.5, 113.1, 115.5, 122.1, 127.3, 127.5, 128.1, 129.2, 130.3, 137.3, 139.3, 145.4, 150.3, 156.6, 157.8, 159.4, 163.1. Elemental analysis. Found. %: C 75.79; H 5.41; N 10.51. C_25_H_21_N_3_O_2_, Calculated, %: C 75.93; H 5.35; N 10.63. 

3-Benzyl-4-imino-5-phenyl-4,5-dihydro-3*H*-chromeno[2,3-*d*]pyrimidin-8-ol (19): IR (KBr): 3340, 3115 cm^-1^. ^1^H N.M.R. (DMSO-d_6_) (ppm) (*J*, Hz): 4.36 (1H, s, H_benzyl_), 4.78 (1H, s, H_pyran_), 6.54-7.47 (13H, m, H_aromatic_), 7.79 (1H, s, NH), 8.51 (1H, s, H_imine_), 9.32 (1H, s, OH). ^13^C N.M.R. (DMSO-d_6_) (ppm): 42.9, 64.4, 121.2, 122.8, 123.7, 134.1, 136.5, 138.2, 140.5, 142.1, 144.3, 145.7, 148.3, 150.6, 152.1, 154.7, 156.7, 158.2, 159.6, 160.4. Elemental analysis. Found, %: C 75.48; H 5.28; N 10.90. C_24_H_19_N_3_O_2_, Calculated, %: C 75.57; H 5.02; N 11.02. 

12-Phenyl-10H-benzo[*f*]chromeno[2,3-*d*]pyrimidine-11(12*H*)-imine (20): IR (KBr): 3437, 3317, 1647 cm^-1^. ^1^H N.M.R. (DMSO-d_6_) (ppm) (*J*, Hz): 6.06 (1H, s, H_pyran_), 7.08-8.24 (14H, m, H_aromatic_, H_imine_, NH). ^13^C N.M.R. (DMSO-d_6_) (ppm): 34.7, 97.6, 118.1, 123.6, 125.3, 127.1, 127.5, 128.1, 128.9, 129.1, 129.8, 131.3, 144.2, 148.3, 156.8, 162.9. Elemental analysis. Found, %: C 77.64; H 4.50; N 12.99. C_21_H_15_N_3_O, Calculated, %: C 77.52; H 4.65; N 12.91. 

*Antibacterial activities*

We used the agar disk diffusion method for this purpose. Each chemically synthesized materials (5 mg) was solved in DMSO as a solvent and 100 μL of known concentration of the test compounds was introduced onto the disks (7 mm) and then allowed to dry. Then the disk was introduced onto the upper layer of the medium with the bacteria. 100 μL of solvent (DMSO) was added to another disk and implanted as a negative control on each plate along with the standard drugs. The plates were incubated overnight at 37 °C. The inhibition zones were measured and compared with the standard drugs. The results are given in Table 2. The inhibition zone numbers are the average of three times of dependent experiments. 

## Results and Discussion

**Scheme 1:**
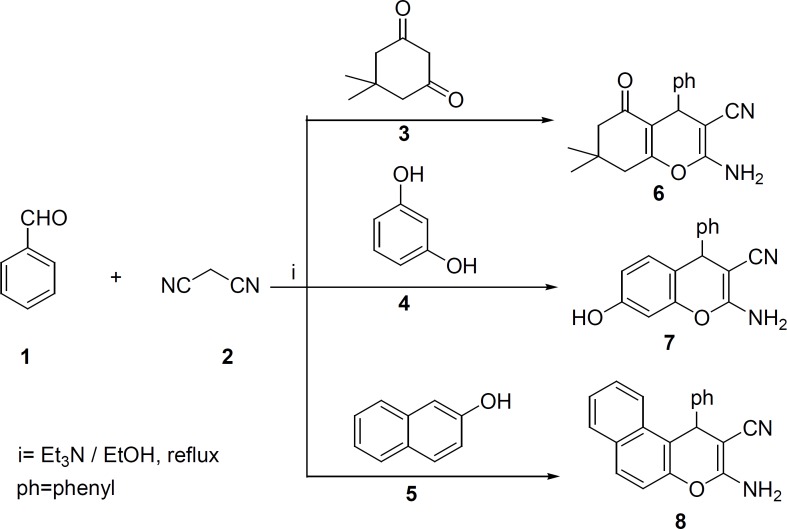
Synthetic pathway of compounds 6-8.

Compounds 6-8 were synthesized according to literature (33-35) (Scheme1). The appearance of the IR absorptions bonds due to the NH2 and CN groups of synthesized compounds 6-8 clearly confirmed the formation of these compounds.

The Imines 9-11 were prepared via reaction of 6-8 with triethylorthoformate in dioxane as a solvent. The IR spectra of 9-11 revealed the absence of the amino group, which is in support of imine formation. In the ^1^H NMR spectra of these compounds, the appearance of triplet and quartet signals at high field is attributed to the resonance of ethoxy group protons. The resonance of imine proton at low field is also a good evidence for formation of imine structures.

**Scheme 2:**
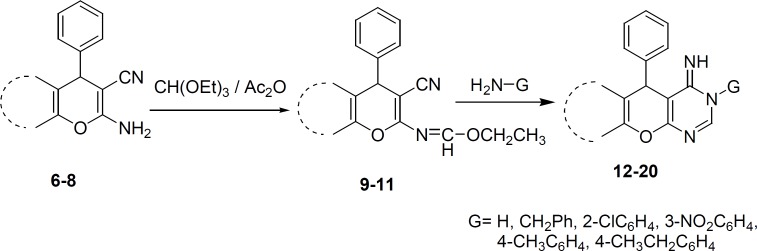
Synthetic pathway of compounds 9-20.

Synthesis of compounds 12-20 was achieved through the reaction of 9-11 with ammonia or primary amine in ethanol or dioxane as a solvent (Scheme 2). In this reaction, when the G group is hydrogen, the reaction may produce two amino or imino tautomers. However, the NMR evidence is consistent with imino form (36), which shows two broad separate signals for two different NH groups. Yield of products after recrstallization from ethanol was in the range of 65%-93% (Table 1). The NMR spectra, as well as the elemental analysis data of these compounds are consistent with the expected structures.

**Table 1:** Synthesis of chromenopyrimidines 12-20

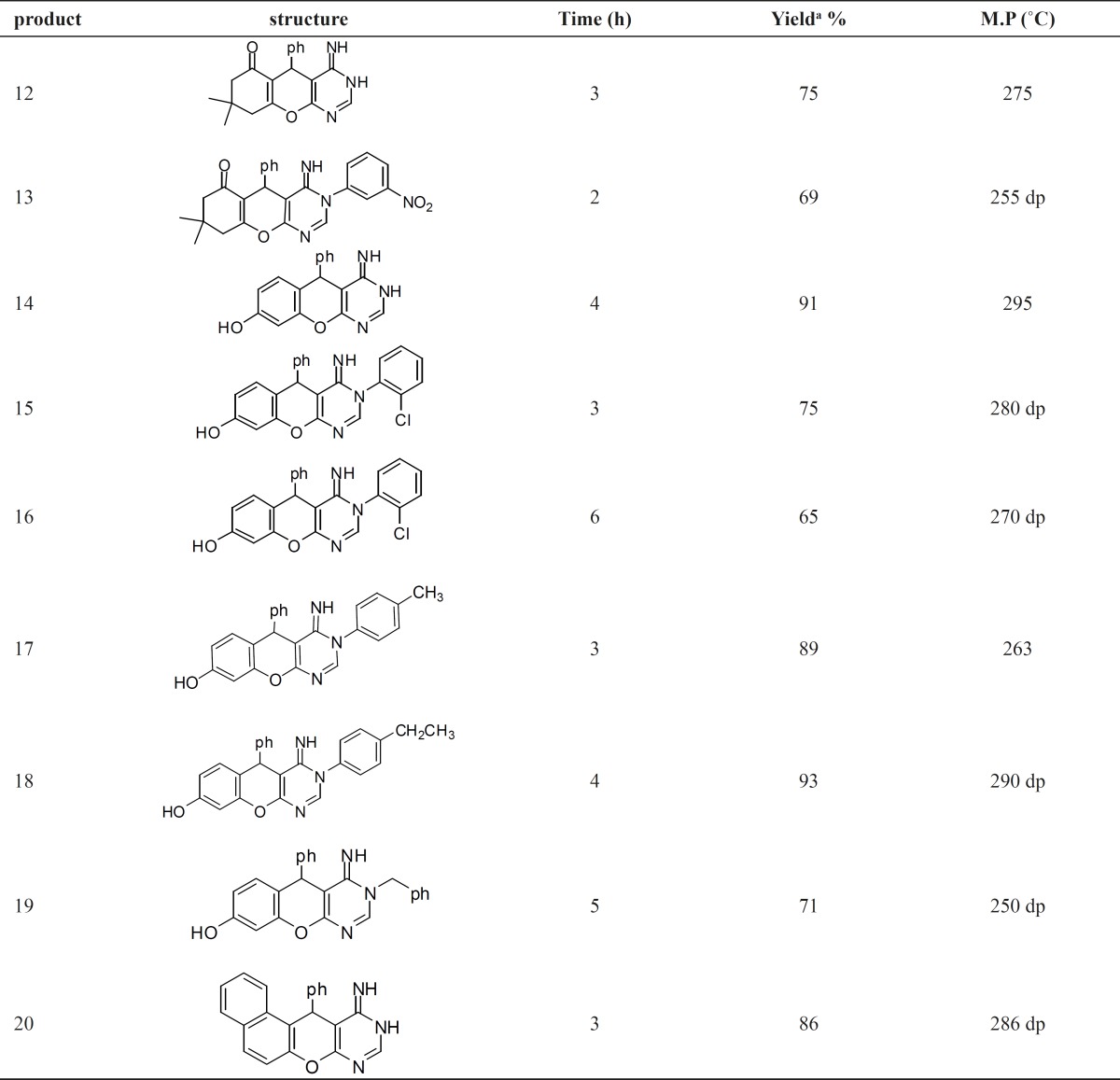

The verification of antibacterial screening data revealed that seven out of nine tested materials showed antibacterial activities against Staphylococcus aureus and Escherichia Coli bacteria (Table 2). The maximum and minimum activities against Staphylococcus aureus were related to materials No. 15 and 18, respectively and maximum and minimum activities against Escherichia Coli were related to materials No. 15 and 16, respectively.

In conclusion, we have synthesized a series of novel chromenopyrimidine derivatives in suitable yields and the biological activity of these materials was also investigated.

**Table 2:** Bacterial inhibition zone around disks containing samples

**Escherichia Coli (mm)**	**Staphylococcus aureus (mm)**	**Compound**
15 ± 0.2 mm	23 ± 0.2 mm	12
10 ± 0.2 mm	20 ± 0.2 mm	13
13 ± 0.1 mm	22 ± 0.2 mm	14
18 ± 0.2 mm	25 ± 0.1 mm	15
9 ± 0.1 mm	21 ± 0.3 mm	16
10±0.1 mm	--	17
17 ± 0.1 mm	18 ± 0.3 mm	18
--	24 ± 0.2 mm	19
14 ± 0.2 mm	--	20
--	--	DMSO
Gentamicin 12 mm	Gentamicin 19 mm	Standard drugs

Indicates resistance of bacteria to compounds.
